# Exam preparatory course for the 2nd part of the German medical examination in obstetrics and gynecology – a potential tool for the recruitment of new residents during the occupational decision process before the practical year?

**DOI:** 10.1186/s12909-019-1457-6

**Published:** 2019-01-17

**Authors:** Fabian Riedel, Maximilian Riedel, Alexander Freis, Joerg Heil, Michael Golatta, Florian Schuetz, Christof Sohn, André Hennigs

**Affiliations:** 1 Department of Gynecology and Obstetrics, Heidelberg University Hospital, Heidelberg, Germany; 2 Department of Gynecology and Obstetrics, Klinikum rechts der Isar, Technical University Munich, Munich, Germany; 3 Department of Gynecologic Endocrinology and Fertility Disorders, Heidelberg University Hospital, Heidelberg, Germany

**Keywords:** Exam preparation course, Obstetrics and gynecology, Recruitment, Occupational decision process

## Abstract

**Background:**

The “Second Stage of the Physician Exam” at the end of the 5th year of medical school in Germany is the final step before the “Practical Year.” An exam preparatory class can cover the complete content of Obstetrics and Gynecology (OB/GYN) in two days. We raise the question of whether such training might promote students’ interest in the given specialty during occupational decision making and whether it could even be used by hospitals as a recruitment tool. This investigation is even more important in the context of fierce competition among young professionals at clinics and in different specialties.

**Methods:**

We conducted a multimodal course evaluation for four exam preparatory courses (each of which lasted two days and involved 8.5 h of teaching), including pre- and post-course tests with 20 multiple-choice questions to quantify the level of skill gain. Additionally, a standardized evaluation of course satisfaction was performed, followed by a post-exam questionnaire that dealt with studying activities and individual professional objectives.

**Results:**

Overall, *n* = 197 students took part in four identical courses. Among them, *n* = 121 completed the pre−/post-course tests, *n* = 170 completed the evaluation, and *n* = 110 completed the post-exam questionnaire. An average improvement from 13.9 to 17.2 correct answers was observed (max. 20; pre−/post-difference 95%-CI: [2.77; 3.86], t-test: *p* < 0.0001). By trend, the students noted that course participation positively influenced their later choice of specialty training (*m* = 3.63; scale 1 = “strongly disagree,” 5 = “strongly agree”).

**Conclusions:**

In addition to self-studying, condensed classroom training is effective and reasonable and might also increase the attractivity of OB/GYN among students and have a positive effect on recruitment.

## Background

Medical students in Germany must pass a nationwide standardized written state examination after their 5th year of medical school in order to enter the final “Practical Year,” which marks the transition between medical studies and future medical occupation and enables them to gain experience in a full-time working environment. This final year is divided into three phases, each of which comprises 16 weeks of training: internal medicine, surgery, and a further practical clinical specialty that can be selected based on personal interests. After completing this year and passing a third state exam (which is practical and verbal), students earn their German Medical License (“Approbation”) [1], and specialty training can begin. Unlike in other countries, Germany has no standardized residency program. Training can be performed at any institution accredited by the local authority and takes around 5–6 years (depending on the specialty). There is no structured application procedure or matching process for residency applicants like in the United Kingdom or the United States [2, 3]: Each aspiring resident applies individually to the head of a hospital department or outpatient institution. In choosing the elective specialty for their final year and determining the institution for this training (which is very flexible – i.e., it can take place at any academic hospital in Germany and even at academic hospitals abroad), many students are aware of the impact of this decision on a successful residency application. The final year traditionally serves the purpose of connecting the medical students with their desired departments and helping them to plan their application process for a future residency. This choice of an elective specialty must be made six months in advance – i.e., during the preparation for the second state exam. This exam is generally regarded as a major obstacle during the entire medical education program in Germany. During three consecutive days, a total of 320 multiple-choice questions (with five possible answers, only one of which is correct) from all specializations of medicine have to be answered and are formed as individual questions or framed as case studies. Many of the questions relate to the field of Obstetrics and Gynecology (OB/GYN). This exam design requires effective preparation. In this context, two developments have been characteristic in recent years regarding individual exam preparation and are the subjects of our study.

First, new studying strategies have become necessary due to the increasing amount of specialty knowledge that has led to new commercial and digital studying platforms, which have become very popular in Germany among medical students in recent years. These platforms include the market leader “AMBOSS” (Miamed Ltd., Cologne, Germany), which is about to enter the US market. Such platforms provide efficient exam preparation as they allow time-saving repetition of medical facts and enable students to test their knowledge with original multiple-choice questions from former exams. The medical facts are compiled and condensed based on their relevance from former exams. Standardized studying schedules, the focus on frequently asked questions, and an individual statistical evaluation aid in students’ efficient preparation [4, 5]. Thus far, there is no evaluation available on the question of how individual exam preparation has changed in recent years due to these new technologies.

Second, both residency applicants and employers have become aware of the beneficial job perspectives. Facing an increasing shortage of medical staff, hospitals today are being forced to fiercely compete for qualified young professionals. This situation is also particularly relevant to entrants to OB/GYN programs [6]. Currently, there are approximately 6000 physicians in training for OB/GYN, with around 580 finishing annually, 80% of whom are women [7]. Nevertheless, it is also difficult for many hospitals to fill vacancies with experienced OG/GYN specialists [6]. As physicians desire a better work-life balance more so today than in the past, hospitals and clinics need to qualify additional medical staff [8, 9]. This situation is further reinforced by an increasing share of part-time jobs [10] and the effects of a growing “feminization” of the candidate pool while general patterns of career planning have not changed significantly among young physicians [11, 12]. As a result of these developments, the question remains as to which factors during medical school education make an impact on choosing a specialty after graduation. Various influencing factors for choosing OB/GYN within different stages of medical training – such as nursing placements [13] or clinical internships [14] – have already been examined. Interestingly, OB/GYN loses potentially interested candidates between the beginning of clinical studies (3rd year) and the final practical year [15]. Studies have revealed that this effect applies to OB/GYN approximately as much as in classical “surgery” specialties – e.g., in orthopedics (OB/GYN -37.2% vs. orthopedics − 41.8%) – which would hypothetically lead to a 19% under-supply of physicians working in this field when compared with the actual percentage of OB/GYN specialists in Germany (who represent around 6.9% of all physicians) [16]. However, these surveys also indicate also that a considerable share of students have not reached a final decision regarding their specialty immediately before finishing their academic training. It would be reasonable to provide students with a better understanding of possible alternatives during the orientation period as their further training in the practical year is determined by two obligatory terms in internal medicine and surgery and another elective term that has to determined during the 5th year [1].

An exam repetition course would provide an excellent opportunity to demonstrate the whole spectrum of the specialty while simultaneously offering additional exam preparation. Because OB/GYN is often not students’ first choice for a residency [17], it is important for OB/GYN to differentiate itself from other specialties in the university teaching program and to offer special incentives, especially toward the end of the medical degree and before students’ decide on a specialization. An exam preparation course might be capable of stimulating undecided students, further encouraging pre-existing interest, and building a valuable network with potential residents. A first evaluation at Heidelberg University Women’s Hospital revealed the feasibility of such a course and its high rate of acceptance by the participants in terms of a “proof-of-concept” study [18]. After the first two pilot courses, four additional courses took place between July 2015 and February 2017. The established course concept was continued with 2 × 8.5 h of classroom teaching covering all exam-relevant topics in OB/GYN, including a joint discussion of 120 representative “original” multiple-choice questions. Thus far, there is no other preparatory course for OB/GYN in Germany, and the impact of such a course in the context of studying strategies and specialty choice remains unclear.

The aim of the following study was to evaluate whether an exam preparatory course in OB/GYN could face two important challenges in the context of the final stage of medical school education: first, new studying strategies and the massively increased distribution of digital studying platforms for state exam preparation (which might make face-to-face studying opportunities redundant) and, second, the fierce competition for motivated OB/GYN residency applicants. The latter challenge leads to questions regarding the factors that potentially influence specialty choice among course participants during the occupational decision process. The question of whether such a short course could – in principle – increase interest and attraction toward specialty training in OB/GYN lies at the center of the present study and – to our knowledge – has not yet been evaluated.

## Methods

### Course description

Each course took place about three months before the state examination. The two-day course was organized by dividing the subjects into two groups (OB/GYN) and then further subdividing them into the following areas: Day 1: general gynecology, endocrinology, gynecological oncology, and urogynecology; Day 2: reproductive medicine, maternal/fetal medicine, obstetrics, gynecological infections, and emergencies. The course was designed and led by residents in OB/GYN with 2–5 years of work experience and first-hand familiarity with the specific demands of the exam. The organization of the course and the compiling of the course material were supported by medical students who had recently passed their exam and who were thus able to consider difficult material and pitfalls. When possible, the course integrated other subjects closely related to OB/GYN, such as pediatrics, infectiology, pharmacology, surgery, and urology. During the course, there was a dynamic variation between lectures and the joint processing of representative multiple-choice exam questions [18].

### Study design

A descriptive study was conducted in the setting of the exam preparation courses offered by Heidelberg University Department of Gynecology and Obstetrics.

#### Course participants and study inclusion criteria

The target audience was students who were about to take the state exam at the Medical Faculties of Heidelberg and Mannheim (which both belong to Heidelberg University, Germany). Course participation was voluntary and was not linked with the study participation described in the following section.

#### Course evaluation and online post-exam questionnaire

To assess the course concept, after students had completed the course, we asked for a voluntary evaluation concerning their general satisfaction with the course set-up and how it was run as well as general questions about studying strategies. The evaluation used written anonymous questionnaires with 42 items and three open questions with free-text reply fields. Sixteen of the 42 items on the course evaluation questionnaire used a 5-point Likert rating scale with which the participants indicated their agreement or disagreement with the statement in the given item (1 = “strongly disagree,” 2 = “disagree,” 3 = “neither agree nor disagree,” 4 = “agree,” 5 = “strongly agree”). The other questions were either dichotomous or classification questions with multiple possible answers. A further item asked for an overall evaluation of the course from 1 (“extraordinarily bad”) to 6 (“extraordinarily good”). The evaluation questionnaire was developed with the support of the Division for Integrative Educational Advising of Heidelberg University Medical School. Established standard items for the evaluation of teaching at the medical school during the regular semester were used but were adapted specifically for this exam preparation course. Furthermore, about one month after the state exam, the students who had participated in the in-course evaluation received an e-mail invitation for an additional post-exam online questionnaire that included 20 dichotomous or classification questions concerning a retrospection of the exam. The evaluations were voluntary.

#### Evaluation of studying progress

To analyze the studying progress, two tests with 20 multiple-choice questions each were administered before and after the course. The questions were analogue to questions from former published state exams (i.e., multiple-choice questions with 5 options and only one correct answer). The reliability of these tests as test instruments was assessed by the Competence Center for Medical Exams in the proof-of-concept courses by means of Cronbach’s α, whereby a Cronbach’s α > 0.7 was set as the threshold for sufficient reliability [18]. There were two versions of the test: Half of the participants received Version 1 at the beginning and Version 2 at the end. The time limit for each test was 30 min – i.e., students had 90 s on average for each question (analogous to a real exam setting). Each correct answer corresponded with 1 point (max. 20 points). Taking both the pre- and post-tests was voluntary.

#### Statistical analysis

The results of the tests at the beginning and end of the course were evaluated with a t-test for paired samples. A *p*-value < 0.05 was defined as statistically significant. The mean and/or relative proportion for each answer was calculated descriptively for the items of the course-evaluation questionnaire. The assessment was conducted via SPSS (IBM, version 22.0). Tables and figures were generated in Excel (Microsoft, version 2016).

## Results

### Course participants

In total, 197 students took part in four courses at Heidelberg Medical School between July 2015 and February 2017. All participants had previously completed curricular content in OB/GYN with a university examination according to the German Medical License Act [19]. Over 90% of the participants intended to complete the state exam at the next possible date – i.e., generally three to four months after the course. Around two-thirds of all participants had taken a semester off for full-time exam preparation. For details, see Table 1.

**Table 1:** Evaluation results for the exam preparatory course in obstetrics and gynecology

General		%
Sex (*n* = 166)	male	33.1
	female	66.9
Semester off before the exam (*n* = 165)	yes	61.2
	no	38.8
Practical experience in GYN/OB (*n* = 169)^a^	nursing placement	12.4
	clinical internship	34.9
	practical year (already finished)	2.4
	elective term in OB/GYN	16.6
	no experience	54.5
Self-assessment in OB/GYN (*n* = 163)	far below average	1.8
	below average	18.4
	average	66.9
	above average	12.3
	far above average	0.6
Specialties		%
Desired profession (*n* = 134)^a^	patient care	90.3
	research	5.2
	second degree	2.2
	private economy	1.5
	not decided yet	5.9
	other	1.5
Desired specialty (*n* = 144)^a^	internal medicine	21.4
	pediatrics	13.1
	OB/GYN	13.1
	surgery	6.2
	orthopedics/trauma	4.2
	general medicine	6.2
	anesthesiology	4.9
	neurology	5.5
	radiology	3.5
	not decided yet	14.5
	other	11.7
Important aspects concerning specialty choice (*n* = 134)^a^	interest in the subject matter	93.3
	work-family balance	64.2
	working hours	39.6
	potential earnings	17.2
	career opportunities	10.4
	other	3.0
How definitive are you concerning your specialty choice? [1 = “not at all,” 5 = “to a high degree”] (*n* = 118)	*m* = 3.93	

*Abbreviations*: *OB/GYN* obstetrics and gynecology, *m* mean

^a^more than 1 choice was possible

Among all participants, *n* = 121 completed the pre- and post-course tests, *n* = 170 completed the evaluation, and *n* = 110 completed the post-exam questionnaire.

### Course evaluation

#### General characteristics

Most of the students were female (66.9%), and more than one-third had already had practical experience in OB/GYN, mostly in clinical electives (34.9%). About half of the students (54.8%) claimed to have no practical experience at all. Two-thirds of all participants expressed average knowledge in OB/GYN (66.9%) in their self-assessment. For details, see Table 1.

#### Specialty choice

Most participants had planned a “classical” career in patient care (90.3%), and most had chosen a path in internal medicine (21.4%), followed by pediatrics and OB/GYN (both 13.1%). Generally speaking, the participants were highly determined in their choice for a specialty (*m* = 3.63; scale 1 = “not at all”; 5 = “to a high degree”). One in seven (14.5%), however, were still completely undecided. For details, see Table 1.

#### Interest in OB/GYN and the impact of course attendance

We also directly asked about the impact of the course on the final choice of a specialty. The results tended toward a positive influence of the course as the interest in OB/GYN rose in nearly one-third of the participants. A disproportionately high (relative) gain in interest was seen after the course, especially in the subgroup with the lowest interest before the course. In this group (17%, i.e., *n* = 24), almost half (48.3%) claimed to have greater interest in OB/GYN afterward (Table 2).

**Table 2:** Interest in obstetrics and gynecology and impact of course attendance

Questions			%
Do you believe that an exam preparatory course or similar offerings could positively influence your choice of a specialty? [1 = “highly disagree,” 5 = “highly agree”] (*n* = 131)	*m* = 3.63		
Interest in OB/GYN before the course (*n* = 135)	low (*n* = 23)		17.0
	moderate (*n* = 60)		44.4
	high (*n* = 52)		38.6
(relative) interest trend in OB/GYN after the course (*n* = 135) [depending on interest group before; in%]	all (*n* = 135)	decreased	0.0
		equal	70.4
		increased	29.6
	low (*n* = 23)	decreased	0.0
		equal	51.7
		increased	48.3
	moderate (*n* = 60)	decreased	0.0
		equal	80.0
		increased	20.0
	high (*n* = 52)	decreased	0.0
		equal	67.3
		increased	32.7
Assessment of the course in general [1 = “extraordinarily bad,” 6 = “extraordinarily good”] (*n* = 166)	*m* = 5.43		

*Abbreviations*: *OB/GYN* obstetrics and gynecology, *m* mean

#### Pre-post course tests

**Fig. 1:**
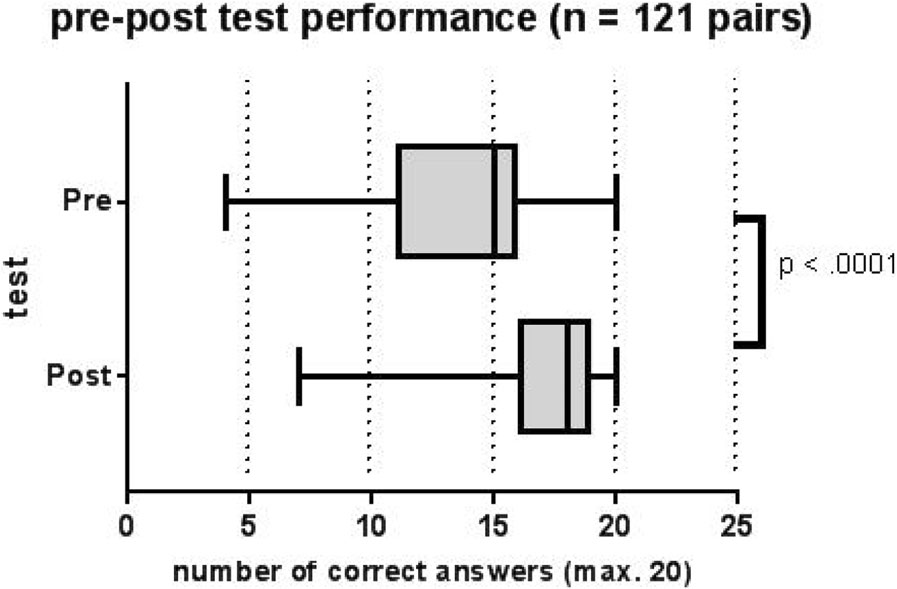
Box-and-whisker plot showing the pre- and post-test performance of four exam preparatory courses in obstetrics and gynecology with a maximum of 20 correct answers (*n* = 121 pairs). Paired t-test *p*-value was calculated

The analysis of 121 fully completed pre- and post-tests demonstrated an improvement in the number of correctly answered multiple-choice questions from 13.9 to 17.2 (max. 20; pre-post-difference: 95%-CI: [2.77; 3.86], t-test: *p* < 0.0001; see Fig. 1).

#### After the exam

The online post-exam questionnaire was completed after an e-mail invitation by 110 former course participants, which reflected a quote of 55.8% of the total number of participants. The results of this post-exam questionnaire are presented below.

### Studying strategies

After the exam, most participants claimed to have needed 100–120 days (76.2%) for exam preparation with six to eight hours of daily studying (44.5%) during six (49.7%) or seven (45.6%) days a week. This amount of studying was sufficient for the majority of participants (71.1%), and the results from the exam for nearly half of the participants (48.2%) confirmed the prior results from the exam preparation.

In terms of studying strategies, almost all students (98.9%) used pre-arranged, standardized studying schemes. Digital studying platforms were the preferred media (87.2%), while studying with literature and with former exam questions was used similarly frequently (54.5 vs. 45.5%). Short textbooks were only used as the primary studying tool by a minority (12.8%), and no student used detailed textbooks or drew on class notes (both 0%). More than half of the students focused solely on the content that had been assigned by the exam authorities in Germany. Overall, for the majority of students (64.4%), the estimated expenses for exam preparation (books, licenses, etc.) were moderate and below 100.00 EUR.

### The exam in retrospective

The “Second Stage of the Physician Exam” is considered a great challenge overall (2.1; Likert-scale 1 = “very high” to 5 = “very low”) even though the relevance for later patient care is considered rather low (2.99; Likert-scale 1 = “very relevant” to 5 = “irrelevant”). About half of the respondents agreed with the statement that the conception of the exam was “partly appropriate” for assessing basic clinical knowledge. The vast majority (80.3%) appraised the course as a reasonable supplement to exam preparation, and for a large proportion of students, the course was more efficient than pure self-studying (2.43; Likert-scale for the statement: equal input as self-studying, 1 = “strongly disagree” to 5 = “strongly agree”).

## Discussion

The results clearly illustrate two things. First, students greatly appreciated the traditional exam preparatory course with classroom teaching in addition to self-studying. Most students made use of digital studying platforms based on publicly accessible former multiple-choice exam questions, which offered efficient preparation for the individual and a statistical evaluation of the studying progress. However, the primary motivation of the participants in the course was to experience an alternative to self-studying and to save time. The desire for more efficient studying methods due to medical students’ high workload has already been described outside of Germany [20].

Objective studying progress over the two days of the course can be quantified by comparing the pre- and post-course test results (see Fig. 1). Sporadic exam preparatory courses were established for the “First Stage of the Physician Exam” [21], as is the case in Heidelberg. Courses similar to this one for other specialties [22] and general medical care exist [23]; however, based on our knowledge and literature research, there has never been another exam preparatory course specifically for OB/GYN in Germany.

The second thing that the results illustrate involves the perception of the specialty among students after participating in the course. This element is relevant when students have to make their first definitive decisions about their desired profession. Even though the questionnaire was completed after the decision for the elective term in the practical year had been made, 14.5% of the participants were still indecisive about their final specialty choice (see Table 1). This finding is in accordance with results from surveys among medical students in Saxony in their 5th year of studies before their practical year [24]; however, the results are substantially meeker than those from students during the practical year [11]. It is therefore reasonable to argue that a decision in favor of or against a specialty is made during this short period of time (3 terms of 16 weeks each).

Almost one-third (29.6%) of the participants had a greater interest in OB/GYN after having participated in the course. Interestingly, a disproportionately high relative gain in interest was seen after the course (48.3%), especially in the subgroup of students with a low interest from the beginning (17.0%). This finding matches with results from Canada and Hong Kong, where a higher proportion of candidates could be attracted via good teaching [25, 26]. A “gender bias” in medical education that restricts male students from certain parts of OB/GYN teaching has been described in other cultural backgrounds [27]. Corresponding data for Germany or Europe are not available.

Most students in Germany do not gain practical experience in OB/GYN aside from the obligatory content within the medical curriculum. Clinical clerkships with full-time experience in a working environment are not mandatory, which is in line with our results. Despite this fact, almost half of our study population had already gained practical experience via individual initiative (by organizing an internship in OB/GYN, etc.). According to these results, interest in the subject matter (93.3%) and a good work-family balance (64.2%) are crucial for most of our participants in the matter of choosing a specialty, as other surveys have demonstrated [28]. Active human resources management and dynamic personal policy in hospitals have become highly relevant as medical positions cannot be adequately filled with candidates due to an increasing share of female doctors who make use of family planning schemes and part-time contracts to maintain a more ideal work-life-balance. OB/GYN has been considerably affected by this development as 85% of the candidates are female and recruitment issues exist for senior physicians [7]. Thus far, recruiting problems have only been described for general medicine in Germany due to the low visibility of the specialty during studies [29]. However, as the academic training takes place in a university setting, the situation of general medicine as a non-university profession is even more aggravated. It is precisely this issue of limited visibility among students that could be successfully addressed via a preparatory course.

Other countries have comparable problems in finding a sufficient number of young professionals for OB/GYN [30]. Concrete ideas for improvement have been proposed [31], but it is necessary to consider each country’s unique educational system as compared with that of Germany, which still grants medical students many freedoms in terms of gaining clinical experience in different fields. Contrary to results from other surveys among medical students, the vast majority of our collective strove for an occupation in patient care (90.3%). A non-clinical profession at this early stage of the career was not an option for most participants.

## Conclusions

Students appreciated a voluntary, condensed, “face-to-face” exam preparatory course for OB/GYN even though digital studying platforms with self-studying make up the largest share of the exam preparation process today. The short-time results demonstrated a significant gain in knowledge among participants. Moreover, such a course could markedly increase the interest of potential candidates in a given profession during the decisive period in which they chose a specialty while still in the final phase of medical education in Germany. This possibility must be seen in the general context that recruiting qualified young medical professionals is increasingly challenging due to fierce competition between clinics as potential employers and between different specialties. Future studies need to explore a possible long-term effect of influencing factors, such as a preparatory course on specialty- or career choice.

## Limitations

The degree to which these results can be generalized is limited. The collective of participants in the course is certainly not representative because it included an above-average share of medical students with an a priori enhanced interest in OB/GYN. This fact is indicated in the large share of students who had already had practical experience or planned an elective term for the practical year in OB/GYN (Table 1). However, the share of female participants (66.9%) was only slightly above the average of female medical students in general in Germany, which is 61.6% (2015) [32], even though they generally display a disproportionately high interest in OB/GYN.

Furthermore, the postulated studying progress derived from the results (one multiple-choice test directly after the completion of the course) is probably not a reliable predictor of medium- or even long-term progress and cannot prognose the final results of the state exam, as was found in our pilot study [18]. It is possible to argue that the primairly short-term memorization of detailed information in the course in fact simulates a realistic setting due to the requirements of the multiple-choice state exam. In general, the state exam results do not report detailed performance in individual clinical disciplines, which makes estimating the objecitve impact of course participation very difficicult. Moreover, due to data privacy protection regulations, it is not possible to compare the course test results with later results in the state exams. Further extensive studies would be needed to evaluate the demand and use of an exam preparation course for students and clinical departments alike, and a mid-term follow-up on the impact of career choice due to course participation would be vital.

## References

[1] German Federal Ministry of Health. Approbationsordnung für Ärzte vom 27. Juni 2002 (letzte Änderung 17. Juli 2017). Bundesgesetzblatt. 2002;1:2405.

[2] Zavlin D, Jubbal KT, Noé JG, Gansbacher B (2017). A comparison of medical education in Germany and the United States: from applying to medical school to the beginnings of residency. Ger Med Sci.

[3] Bogdanyova S, Lermann J, de Sousa Mendes M, Schott S (2015). Becoming a resident in Germany: an experience-based practical guideline. Arch Gynecol Obstet.

[4] Obst O, Salewsky V (2013). How do today’s students learn? An e-book study of the branch library of medicine, University of Münster. GMS Med Bibl Inf.

[5] Abrusch J, Marienhagen J, Böckers A, Gerhardt-Szép S (2015). Quality management of eLearning for medical education: current situation and outlook. GMS J Med Educ.

[6] Mallmann P (2017). Das Nachwuchsproblem in unserem Fach. Frauenarzt.

[7] Riepen T, Mobus V, Kullmer U, Tinneberg HR, Munstedt K (2013). Male and female physicians in hospital gynaecology departments—analysis of the impact of “feminisation” from the viewpoint of medical directors. Geburtshilfe Frauenheilkd.

[8] Gibis B, Heinz A, Jacob R, Muller CH (2012). The career expectations of medical students: findings of a nationwide survey in Germany. Dtsch Arztebl Int.

[9] Schmidt CE, Moller J, Schmidt K, Gerbershagen MU, Wappler F, Limmroth V (2011). Generation Y: recruitment, retention and development. Anaesthesist.

[10] Schott S, Lermann J, Eismann S, Neimann J, Knabl J (2017). Part-time employment of gynecologists and obstetricians: a sub-group analysis of a Germany-wide survey of residents. Arch Gynecol Obstet.

[11] Gedrose B, Wonneberger C, Junger J, Robra BP, Schmidt A, Stosch C (2012). Do female medical graduates have different views on professional work and workload compared to their male colleagues? Results of a multicenter postal survey in Germany. Dtsch Med Wochenschr.

[12] Birck S, Gedrose B, Robra BP, Schmidt A, Schultz JH, Stosch C (2014). Stability of long-term professional objectives of young physicians during postgraduate training. Results of a multicenter cohort study. Dtsch Med Wochenschr.

[13] Kasch R, Baum P, Dokter M, Zygmunt M, Wirkner J, Lange A (2015). Nursing practicum in gynaecology and obstetrics - early influence possibilities for a specialty. Geburtshilfe Frauenheilkd.

[14] Hammoud MM, Stansfield RB, Katz NT, Dugoff L, McCarthy J, White CB (2006). The effect of the obstetrics and gynecology clerkship on students’ interest in a career in obstetrics and gynecology. Am J Obstet Gynecol.

[15] Gotz K, Miksch A, Hermann K, Loh A, Kiolbassa K, Joos S (2011). Aspirations of medical students: “planning for a secure career” - results of an online-survey among students at five medical schools in Germany. Dtsch Med Wochenschr.

[16] Heinz A, Jacob R (2012). Medical students and their career choices. Preferred specialty, where and how to work. Bundesgesundheitsblatt, Gesundheitsforschung, Gesundheitsschutz.

[17] Schmidt K, Meyer J, Liebeneiner J, Schmidt CE, Huttenbrink KB (2012). Generation Y in ENT: leading a young generation of doctors. HNO.

[18] Riedel F, Fremd C, Tabatabai P, Smetanay K, Doster A, Heil J, et al. Exam preparation course in obstetrics and gynecology for the German Medical State Examination: proof of concept and implications for the recruitment of future residents. Arch Gynecol Obstet. 2016;294(6):1235-41. 10.1007/s00404-016-4168-9 27506659

[19] Steiner T, Jünger J, Schmidt J, Bardenheuer H, Kirschfink M, Kadmon M (2003). HEICUMED: Heidelberger Curriculum Medicinale – Ein modularer Reformstudiengang zur Umsetzung der neuen Approbationsordnung. Med Ausbild.

[20] Bloomfield L, Harris P, Hughes C (2003). What do students want? The types of learning activities preferred by final year medical students. Med Educ.

[21] Rengier F, Rauch PJ, Partovi S, Kirsch J, Nawrotzki R (2010). A three-day anatomy revision course taught by senior peers effectively prepares junior students for their national anatomy exam. Ann Anat.

[22] Kühn J, Jabs WJ (2007). The Luebeck compact revision course in Internal Medicine - a pilot scheme in preparation for the amended second state examination in human medicine. GMS J Med Educ.

[23] Spura A, Werwick K, Feissel A, Gottschalk M, Winkler-Stuck K, Robra BP (2016). Preparation courses for medical clerkships and the final clinical internship in medical education - The Magdeburg Curriculum for Healthcare Competence. GMS J Med Educ.

[24] Bartels A, Voigt K, Riemenschneider H, Nitschke-Bertaud M, Bergmann A. Preferred Medical Specialties of Medical Students in Contrast to the Need for General Practitioners in Saxony. Gesundheitswesen. 2017;79(3):188-94. 10.1055/s-0042-102339 27077318

[25] Bedard MJ, Berthiaume S, Beaulieu MD, Leclerc C (2006). Factors influencing the decision to practise obstetrics among Quebec medical students: a survey. J Obstet Gynaecol Can.

[26] Lam CY, Cheung CS, Hui AS (2016). Factors influencing the career interest of medical graduates in obstetrics and gynaecology in Hong Kong: a cross-sectional questionnaire survey. Hong Kong Med J.

[27] Zahid AZ, Ismail Z, Abdullah B, Daud S (2015). Gender bias in training of medical students in obstetrics and gynaecology: a myth or reality?. Eur J Obstet Gynecol Reprod Biol.

[28] Hancke K, Igl W, Toth B, Buhren A, Ditsch N, Kreienberg R (2014). Work-life balance of German gynecologists: a web-based survey on satisfaction with work and private life. Arch Gynecol Obstet.

[29] Steinhauser J, Miksch A, Hermann K, Joos S, Loh A, Gotz K (2013). What do medical students think of family medicine? Results of an online cross-sectional study in the federal state of Baden-Wuerttemberg. Dtsch Med Wochenschr.

[30] Turner G, Lambert TW, Goldacre MJ, Barlow D (2006). Career choices for obstetrics and gynaecology: national surveys of graduates of 1974-2002 from UK medical schools. BJOG.

[31] Bonnett TJ, Roberts AL, Farrell TA (2012). Translating obstetrics and gynaecology undergraduate experience into career aspiration: an audit of Royal College of Obstetricians and Gynaecologists (RCOG) medical student placement standards. J Obstet Gynaecol.

[32] Statistisches Bundesamt (2015). Studierende im Studienfach Medizin.

